# The Asymmetric A^3^(Aldehyde–Alkyne–Amine) Coupling: Highly Enantioselective Access to Propargylamines

**DOI:** 10.3390/molecules24071216

**Published:** 2019-03-28

**Authors:** Jia-Nan Mo, Junqi Su, Jiannan Zhao

**Affiliations:** Zhang Dayu School of Chemistry, Dalian University of Technology, Dalian 116024, China; mojn15@lzu.edu.cn (J.-N.M.); sujunqi@mail.dlut.edu.cn (J.S.)

**Keywords:** aldehydes, alkynes, amines, asymmetric catalysis, copper, multicomponent reactions, nitrogen heterocycles

## Abstract

The recent developments in asymmetric A^3^ (aldehyde–alkyne–amine) coupling has been summarized in this review. Several interesting modifications of the ligands enabled the highly enantioselective synthesis of chiral propargylamines, which are further used in the construction of nitrogen-containing chiral building blocks.

## 1. Introduction

Multicomponent reactions (MCRs), defined as one-pot processes in which three or more accessible starting materials are incorporated into a single product without isolating the intermediates, have gained importance in academia and industry [1,2,3,4]. Multicomponent reactions provide numbers of valuable conceptual and synthetic advantages such as sustainability, operational simplicity, and high convergence. For these reasons, there is no doubt that MCRs are important cornerstones in the diversity-oriented synthesis and combinatorial synthesis compared with stepwise sequential approaches towards complex and important moleculars. By virtue of its inherent advantages, multicomponent reactions have been extensively applied to the total synthesis of complex moleculars and the creation of chemical libraries of drug-like compound [5,6,7]. The past decades have also witnessed a rapid development of asymmetric multicomponent reactions (AMCRs) which can serve as a versatile approach to prepare complex optically pure molecules from simple and readily available achiral substrates.

**Figure 1:**
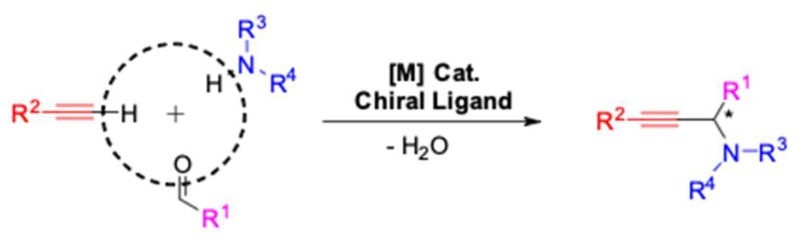
Asymmetric A^3^-coupling reaction.

Over the past several decades, asymmetric multicomponent processes, including the powerful asymmetric A^3^ (aldehyde–alkyne–amine) coupling reactions, have garnered a lot of attention (Figure 1). With this strategy the optically active propargylic amines can be achieved, which are important building blocks for the synthesis of various nitrogen containing compounds, biologically active materials [8], and natural products [9]. Topics on A^3^-coupling have been reviewed previously but predate recent important advances [10,11,12]. Especially, a comprehensive review about asymmetric A^3^-coupling (AA^3^-coupling) has not been reported. The aim of this review is to summarize and discuss recent development in the field of AA^3^-coupling since the first AA^3^-coupling was achieved by Li in 2002 [13]. This article will be divided in two sections based on properties of amines.

## 2. AA^3^ –Coupling Derived from Primary Amine

**Figure 2:**
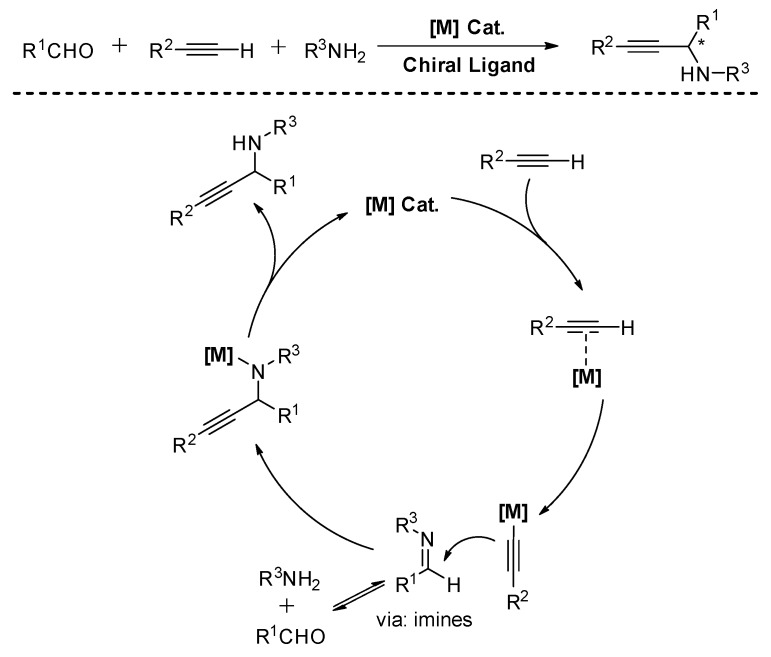
Generalized mechanism of A^3^-coupling with primary amines.

The proposed mechanism for A^3^-coupling involves C-H activation by late transition metals. These metals are well known to form π-metal-complex with terminal alkynes, thereby making the alkyne proton more acidic for further abstraction. After the C-H bond is activated, the presented amines in the reaction media as the weak base could deprotonate the terminal alkynes and generate the desired organometallic alkynyl nucleophile. The imine or iminium ion reacts with the metal acetylide, resulting in the formation of the propargylamines with concomitant regeneration of the metal catalyst for another reaction cycle (Figure 2). In this process, the use of chiral ligands often produces excellent stereoselectivity.

**Figure 3:**
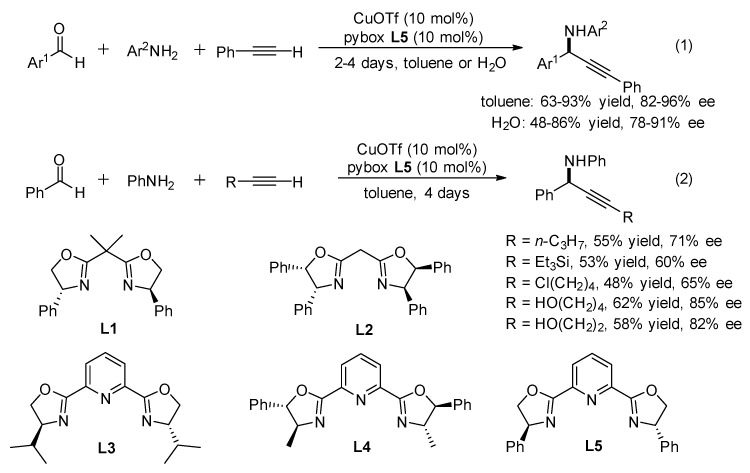
Enantioselective three-component reaction with box and pybox ligands.

The catalytic enantioselective synthesis of propargylamines is derived from primary amines and aldehydes commonly involved in imines as intermediates, and utilizes copper(I)/pybox ligands as the catalytic system. The first asymmetric three-component coupling of primary amines, aromatic aldehydes and aryl alkynes was described by Li in 2002 (Figure 3) [13,14]. The reactions were carried out in organic solvent as well as in aqueous media by utilizing CuOTf as the catalyst. The efficiency of several box (**L1** and **L2)** and pybox (**L3**–**L5**) ligands was explored. Finally, the optimal reaction condition was obtained with CuOTf–**L5**, leading to the corresponding propargylic amines in up to 93% yields and 80–96% ee albeit in low reactivity (2–4 days, Figure 3, Equation (1)). This catalytic system could also be extended to aliphatic alkynes to afford the corresponding propargylic amines (Figure 3, Equation (2)). Unfortunately, however, the enantioselectivity of these reactions is lower, probably due to the less steric hindrance of the alkyl-substituted alkynes.

**Figure 4:**
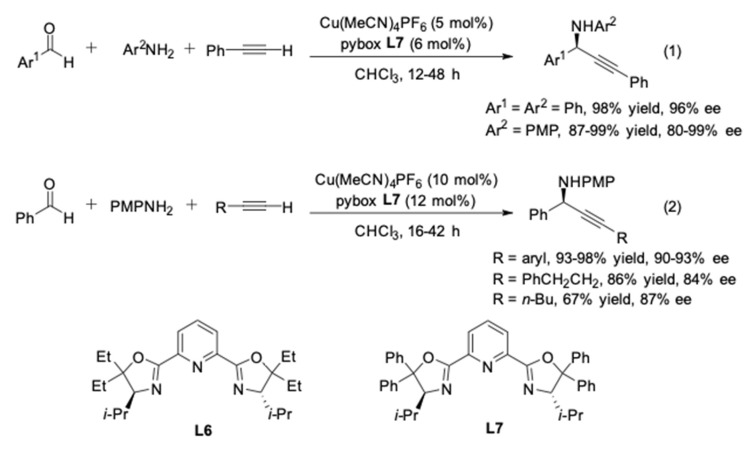
Cu(I)/*i*-Pr-pybox-diPh-catalyzed AA^3^-couplings.

A great number of further pioneering contributions were made by the group of Singh, who improved the efficiency of these coupling reactions with a more suitable ligand [15]. They introduced a *gem* disubstitution at C-5 position of the oxazoline rings (Figure 4, **L6** and **L7**). The outcome of the experiment clearly indicated that the copper(I) complex of *i-*Pr-pybox-diPh **L7** had a drastic effect in enhancing the ee and reducing the reaction time (12–48 h). The one-pot reaction with aniline provided the corresponding product with excellent yield (98%) and enantiomeric excess (96%). The scope of the anilines was also extended to *p*-methoxyphenyl (PMP) amines, which could be oxidatively removed (Figure 4, Equation (2)) [16,17]. However, the three-component reaction with alkyl-substituted alkynes remains unsolved, affording propargylic amines with lower ee (84–87%) compared to aryl-substituted alkynes (Figure 4, Equation (2)).

Encouraged by these results, a variety of *gem*-disubstitued pybox-diPh ligands were prepared from naturally occurring α-amino acids by their earlier reported procedures [18,19,20,21]. As shown in Table 1, the substituent at C-4 chiral center of the oxazoline core had a great influence on the results [22]. Both ligands **L7** and **L8** were superior to all other ligands in stereocontrol. Pleasingly, the enantioselectivity was slightly increased by changing *i*-Pr group to *s*-Bu group (Table 1, entry 2 vs. 1).

**Figure 5:**
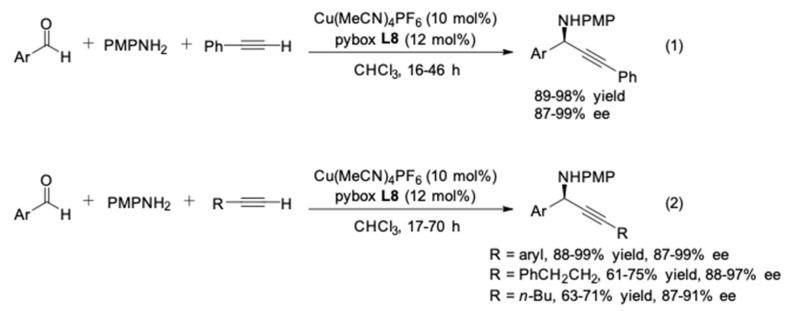
Cu(I)/*s*-Bu-pybox-diPh-catalyzed AA^3^-couplings.

With the optimal ligand **L8**, the enantioselectivity of the reactions with PMPNH_2_ and aliphatic alkynes was also improved, probably due to increased steric hindrance at β-position of the ligand (Figure 5). However, aliphatic aldehyde was not tolerated in this catalyst system.

**Figure 6:**
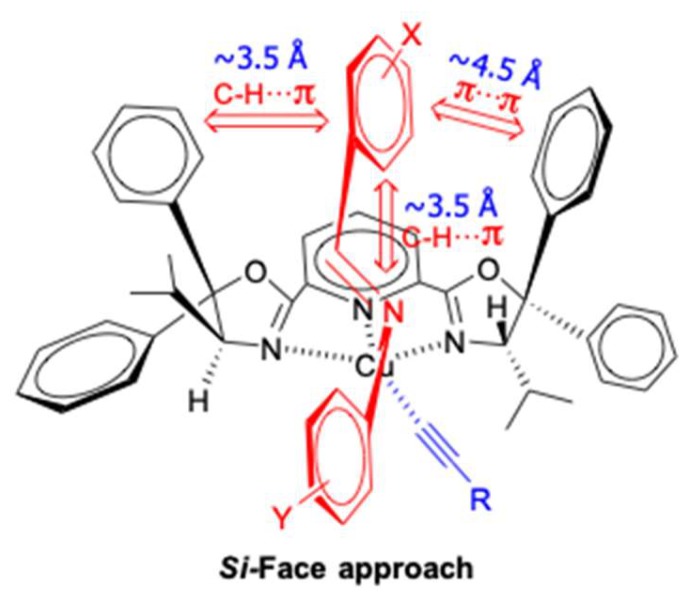
Proposed favored transition state.

Based on the experimental investigations, Singh and co-workers proposed a transition-state model to explain the stereochemical outcome of reaction (Figure 6). It is believed that the ‘N’ of the imine prefers to chelate with the Cu–complex in a manner where there are three stabilizing π-interactions; two C-H···π and one π···π. The aryl group on the sp^2^ carbon of the imine could orient itself in a manner that it is orthogonal and parallel to the phenyl rings in the oxazoline core, respectively. Thus, the transition state becomes highly organized. The steric effect also plays an important role in the reaction. As shown in Figure 6, the *Re*-face of the aldimine is shielded by the β-alkyl group on the chiral carbon atom of the left oxazoline ring. Therefore, the alkynylide attacks from the *Si*-face predominantly to afford the corresponding *R*-propargylamine.

**Figure 7:**
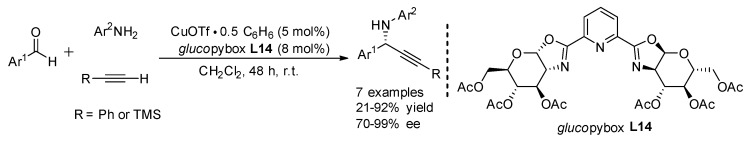
Cu(I)/*gluco*Pybox-catalyzed AA^3^-couplings.

In 2008, Boysen’s group developed a new pybox ligand derived from naturally occurring precursors [23,24,25]. Starting from d-glucosamine, the ligand *gluco*pybox **L14** (Figure 7) could be prepared in four steps. Then the copper(I) complex of **L14** was evaluated in asymmetric A^3^ coupling reactions. The reaction of benzaldehyde and aniline in presence of phenylacetylene was efficiently catalyzed to afford the adduct in 69% yield and excellent 99% ee. For a broad application of the reaction, silylacetylene was used leading to the silylated propargylamines, which could be deprotected to yield terminal alkynes that are very attractive substrates to subsequence transformations. In Boysen’s work, the enantiomeric excess for the reaction with silylacetylene was improved to 90%, which was the best result compared to the previous work. Although the reactivity of the catalyst system need to be further improved, the carbohydrate-based pybox ligand showed great promise as an alternative to conventional pybox ligands due to the inexpensive raw materials.

**Figure 8:**
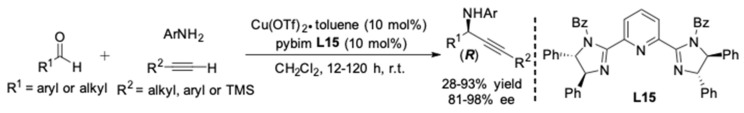
Cu(II)/pybim-catalyzed AA^3^-couplings.

Although the protocols reported by Li, Singh and Boysen provided direct accesses to a range of optically active propargylamines, all of them possess several substrate scope limitations and the three-component reaction with aliphatic alkynes remains unsolved. This problem was identified by Nakamura and co-workers [26,27,28,29,30,31]. In 2008, they developed a chiral Cu(II)-bis(imidazoline) complexes as highly tunable chiral catalysts [32]. In the presence of pybim **L15**, a wide range of alkyl-substituted alkynes was suitable substrates for the reaction, with excellent enantioselectivity observed in most cases (Figure 8). Alkyl aldehyde could also participate in the reaction to afford the secondary propargylic amine in 81% ee albeit in low reactivity (108 h, 39% yield).

**Figure 9:**
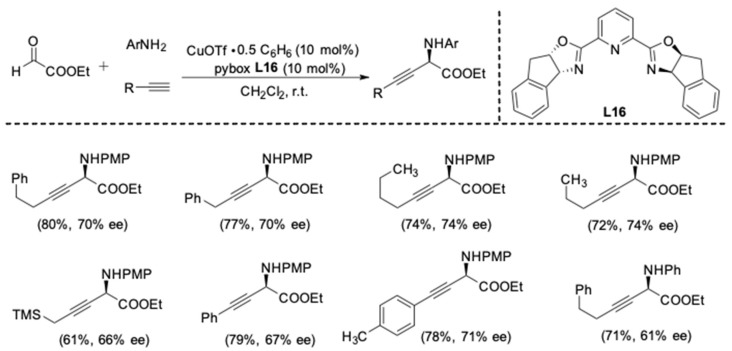
Asymmetric three-component reactions of ethyl glyoxylate.

In 2009, the substrate scope of the aldehydes was extended to ethyl glyoxylate by Shao and Chan [33]. Moreover, the resulted β,γ-alkynyl α-amino acid derivatives were versatile synthetic intermediates [34,35], which enabled facile transformations into a range of unnatural amino acids. The combination of Cu(I) triflate and pybox **L16** was chosen to provide the desired product in good yields and 61–74% ee (Figure 9). The protocol was applicable to both aliphatic and aromatic alkynes.

**Figure 10:**
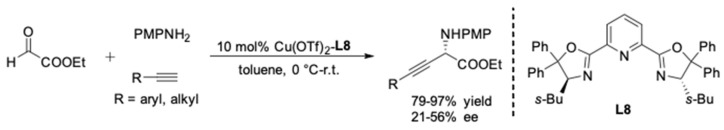
Cu(II)/*s*-Bu-pybox-diPh-catalyzed AA^3^-couplings.

The reactivity of the same reaction could be improved in the catalyst system developed by Singh [22]. The yields were improved to 79–97%, when a complex of Cu(OTf)_2_-**L8** in toluene was used. However, the reaction could only afford the β,γ-alkynyl α-amino acid ethyl esters in poor to moderate ee (20–56%, Figure 10).

**Figure 11:**
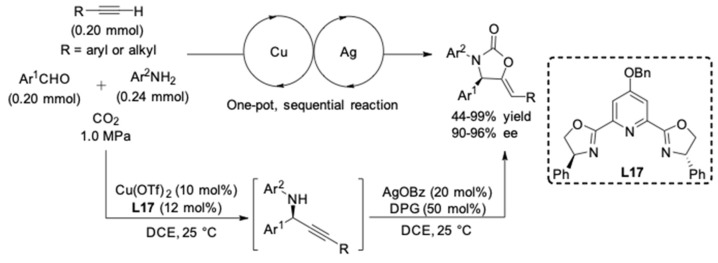
Tandem AA^3^-coupling-carboxylative cyclization.

In 2017, Zhou and colleague reported a tandem AA^3^-coupling-carboxylative cyclization to produce chiral oxazolidinones containing an exocyclic double bond [36]. The reactions were typically performed with phenylacetylene, aromatic aldehydes and anilines employing Cu(OTf)_2_/**L17** to provide the propargylamines (Figure 11). The sequential carboxylative cyclization was catalyzed by AgOBz with a CO_2_ pressure of 1.0 MPa. The one-pot reaction afforded the corresponding chiral oxazolidinones in good yields (up to 98%) and high enantioselectivities (90–96% ee).

## 3. AA^3^–Coupling Derived from Secondary Amine

**Figure 12:**
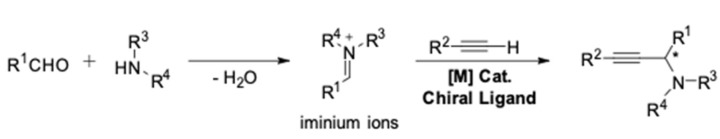
Asymmetric A^3^-couplings with secondary amines.

When secondary rather than primary amines were employed in the AA^3^-coupling reactions, the nature of the reaction changed dramatically. In general, the A^3^-coupling with secondary amines is relatively more reactive, since the iminium ions derived from secondary amines are much more electrophilic than the (neutral) imine counterparts (Figure 12). Again, copper(I) complexes are the most extensively utilized catalysts in this area. However, the reactions with secondary amines require the use of another type of P,N-ligand.

**Figure 13:**
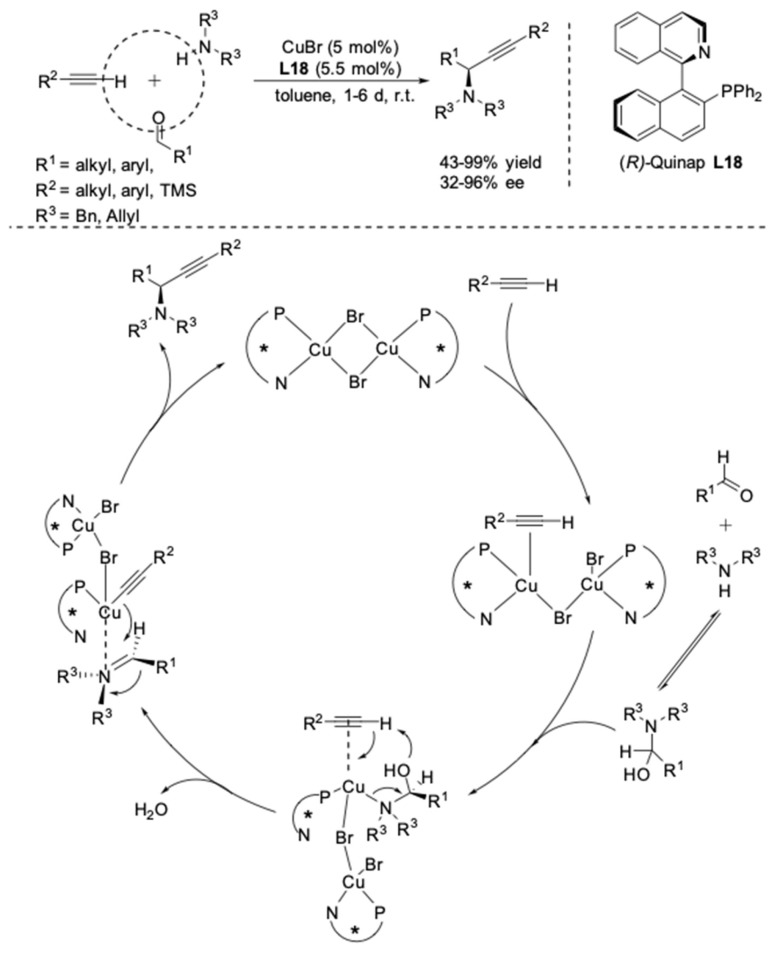
CuBr-Quinap-catalyzed AA^3^-couplings.

For the synthesis of tertiary propargylic amines, the first asymmetric three-component coupling was described by Knochel in 2003. The reactions between secondary amines, aldehydes and alkynes were performed in toluene with CuBr and chiral (2-phosphino-1-naphthyl) isoquinoline (Quinap) ligand **L18** (Figure 13) [37,38]. Both dibenzyl and diallylamines were successfully utilized to afford the optically active propargylamines with high yields and enantioselectivities in most cases. It was found that the employment of aliphatic aldehydes resulted in better enantioselectivities (82–96% ee) than that with aromatic aldehydes (32–78% ee). The alkyne can bear either an aryl substituent or an alkyl substituent. Pleasingly, the highest enantioselectivities were obtained with trimethylsilylacetylene (92–96% ee). According to the strong positive nonlinear effect and the crystal structure of the [CuBr{(*R*)-quinap}]_2_ complex [39,40], it is believed that a dimeric Cu-Quinap complex is the catalytically active species. On the basis of these investigations, a plausible mechanism was suggested by the authors (Figure 13).

**Figure 14:**
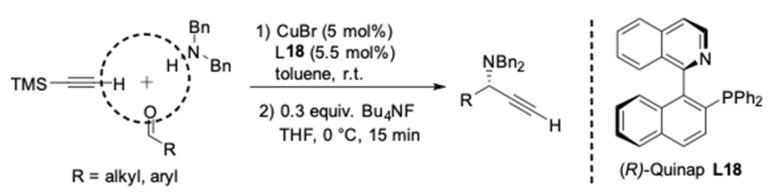
Asymmetric A^3^-couplings and desilylation to terminal alkynes.

To evaluate the synthetic potential of this catalyst system, Knochel^’^s group performed a more profound investigation of their asymmetric A^3^-coupling procedure with trimethylsilylacetylene. The trimethylsilyl group was easily removed by treatment of the silylated propargylamines with Bu_4_NF in THF, leading to the corresponding terminal propargylic amines without a loss of enantiopurity (Figure 14) [41,42].

**Figure 15:**
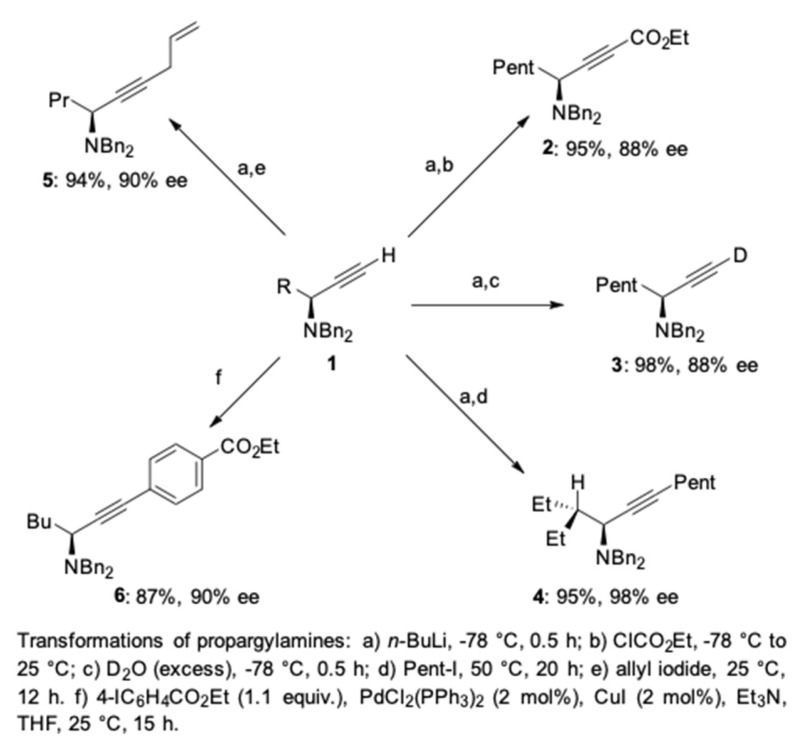
Functionalization of the terminal alkynes.

Further transformations of the terminal alkynes to various functionalized derivatives were also examined (Figure 15) [41]. First, the alkynyllithium reagents, derived from *n*-butyllithium and the terminal propargylic amines **1**, could react with a series of electrophilic reagents (Figure 15). For example, the reaction of the alkynyllithium with ethyl chloroformate provided the chiral alkynyl ester **2** in 95% yield and 88% ee. The deuteration of the alkynyllithium with D_2_O provided the monodeuterated alkyne **3** in 98% yield and >95% deuterium incorporation. Similarly, the alkylation and allylation of the alkynyllithium with pentyl iodide and allyl iodide furnished the corresponding chiral propargylamines **4** and **5** in 95% and 94% yield, respectively. The Sonogashira cross-coupling of the terminal propargylic amines with ethyl 4-iodobenzoate afforded the corresponding phenylacetylene derivative **6** in 87% yield and 90% ee.

Since the catalyst is not soluble in pentane, the Cu(I)-Quinap complex could be recovered by simple filtration and used in up to three times [41]. The results showed that the coupling reaction could be performed with the same catalyst without significant loss in enantioselectivity and with only a slight drop in yield (Table 2).

**Figure 16:**
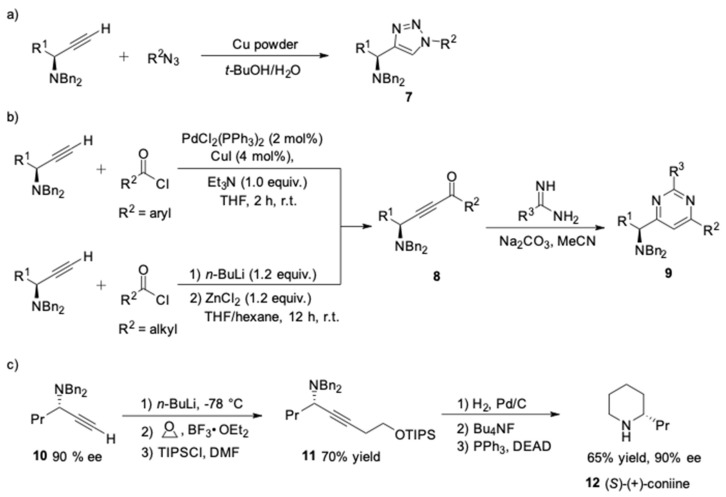
Transformations of the terminal alkynes to heterocycles.

Manipulations of the product also offer opportunities to generate other heterocycles [43,44]. For instance, the terminal alkynes could be efficiently converted, through click reaction [45,46], into useful chiral α-aminoalkyl-1,2,3-triazoles of type **7** in the presence of copper powder (Figure 16a). On the other hand, the terminal alkynes could be acylated resulting in the formation of alkynones **8** in excellent yield [47,48]. Then, treatment of **8** with amidine in refluxing acetonitrile afforded the chiral aminopyrimidines of type **9** without a loss of enantiopurity (Figure 16b) [49,50,51]. Knochel et al. also demonstrated a short enantioselective synthesis of (*S*)-(+)-coniine **12** (Figure 16c), which is a highly toxic alkaloid inducing curare type paralysis [52,53,54]. Alkylation and protection of the terminal alkyne **10** with ethylene oxide and TIPSCl gave the propargylamines **11** in 70% yield. The deprotection of the tertiary amine and reduction of the triple bond were readily achieved by hydrogenation in methanol. Subsequent desilylation and intramolecular Mitsunobu reaction provided an enantioselective access to (*S*)-(+)-coniine **12**.

**Figure 17:**
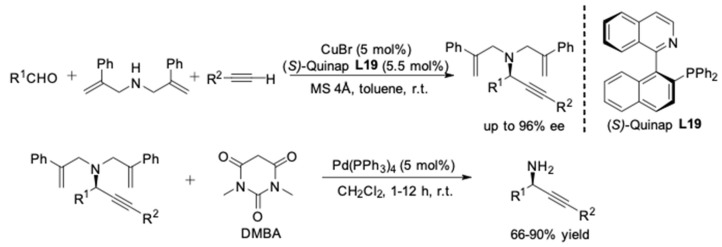
Asymmetric A^3^-couplings and deprotection to primary amines.

In the course of their previous research, it was observed that the use of dibenzylamine is essential to ensure high enantioselectivities. Considering deprotection of the propargylamines would extend their utility, it is highly desirable to deprotect the adduct under mild conditions and yield the corresponding primary propargylamine selectively. However, removal of the benzyl group via hydrogenolysis was accompanied by reduction of the triple-bond. Later, bis(phenallyl)amine was successfully employed as the amine component to furnish bis(phenallyl)-protected propargylamines (up to 96% ee) [55]. Then the allyl group was selectively removed through Pd(0)-catalyzed allylic substitution with 1,3-dimethylbarbituric acid (DMBA), delivering the primary propargylamines in good to high yields (Figure 17).

**Figure 18:**
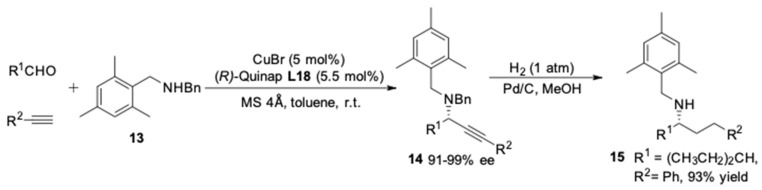
Asymmetric A^3^-couplings of bulky secondary amine.

As the enantioselectivity of the A^3^-coulping reactions was found to be highly dependent on the amine component, the influence of further substituents in dibenzylamine was investigated by Knochel and co-workers [56]. The utilization of bulkier secondary amine, (mesitylmethyl)benzylamine **13**, led to the formation of propargylamines **14** in excellent enantioselectivities (91–99% ee). Furthermore, selective mono-deprotection of the propargylamines was achieved by hydrogenation affording the secondary amine **15** in high yield (Figure 18).

A drawback of Knochel’s protocol is the use of Quinap, which is rather expensive and difficult to prepare. In 2004, Carreira and co-workers creatively designed a new class of P,N-ligands **L20** and **L21** (Pinap) [57]. They were conveniently resolved and prepared in four steps. Both diastereomeric Pinap ligands could be obtained after separation by crystallization or silica gel chromatography. Then the Pinap ligands were tested in Cu-catalyzed AA^3^-coupling reactions. As shown in Table 3, the new ligand showed parallel reactivity to Quinap. Moreover, Carreira et al. observed that the Cu(I) complexes of **L20** and **L21** catalyzed the formation of the propargylic amines in 90–99% ee, which is superior to Quinap when alkyl aldehydes are used.

**Figure 19:**
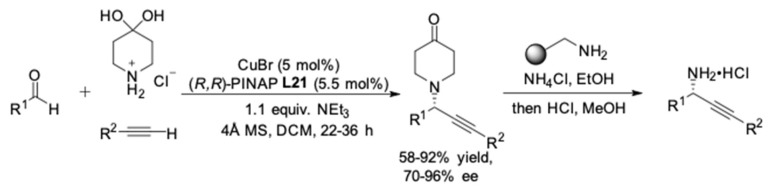
AA^3^-couplings of 4-piperidone·HCl.

Following the success of this protocol, Carreira and co-workers exhibited the AA^3^ coupling reactions with 4-piperidone as the amine component (Figure 19) [58]. Again, (*R,R*)-Pinap **L21** was chosen as the optimal ligand. It is worth pointing out that the reaction rate was increased (up to 5-fold) when dichloromethane was used instead of toluene, since it was observed that the complex formed between CuBr and (*R,R*)-Pinap **L21** could completely soluble in dichloromethane. Carrying out these reactions with silyl-substituted alkynes resulted in higher enantioselectivity (85–96% ee) compared to aryl-substituted alkynes (83–85% ee) and aliphatic alkynes (70% ee). With respect to practically, the tertiary amine adducts could be selectively removed in the presence of a solid-supported amine scavenger.

**Figure 20:**
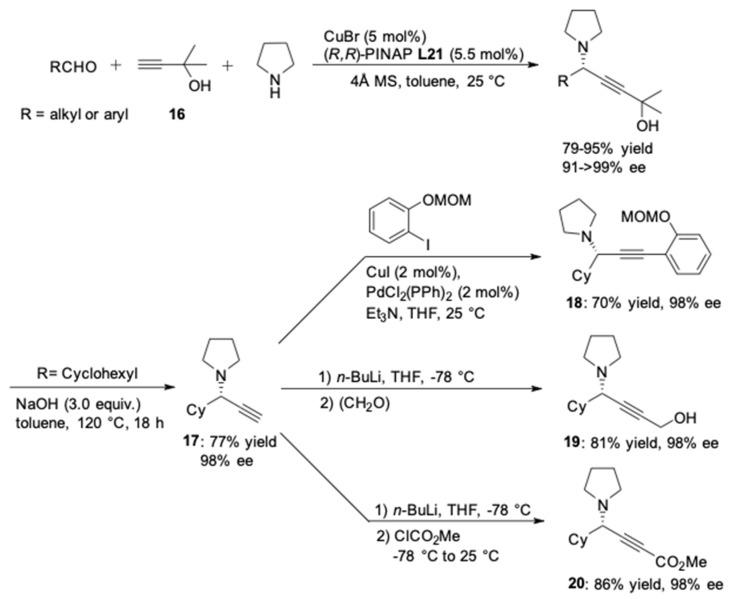
AA^3^-couplings utilizing pyrrolidine as the secondary amine.

On the basis of the previous work, Ma’s group reported a CuBr/Pinap-catalyzed AA^3^-coupling reaction utilizing pyrrolidine or 1,2,3,4-tetrahydroisoquinoline as the secondary amine (Figure 20) [59]. In the presence of 2-methylbut-3-yn-2-ol **16**, the corresponding chiral propargylamines were obtained in high yields (79–95%) and excellent ee (91–>99%). According to the control experiments, the dimethylcarbinol unit in the alkyne component played an important role in ensuring high enantioselectivity. It was believed that the possible coordination of the hydroxyl oxygen to copper(I) was responsible for the excellent stereoselectivity. Furthermore, the 2-hydroxy-2-propyl group could be easily removed to afford the terminal propargylic amines **17** [60]. The generated terminal alkynes could further be functionalized via Sonogashira cross-coupling and furnished the phenylacetylene derivative **18** in 70% yield [61,62]. Treatment of **17** with *n*-BuLi could generate the alkynyllithium intermediate. The following alkylation with paraformaldehyde or methyl chloroformate provided the corresponding alcohol **19** or alkynoate **20** [63], respectively. It is important to stress that both aliphatic and aromatic aldehydes were tolerated in this protocol.

**Figure 21:**
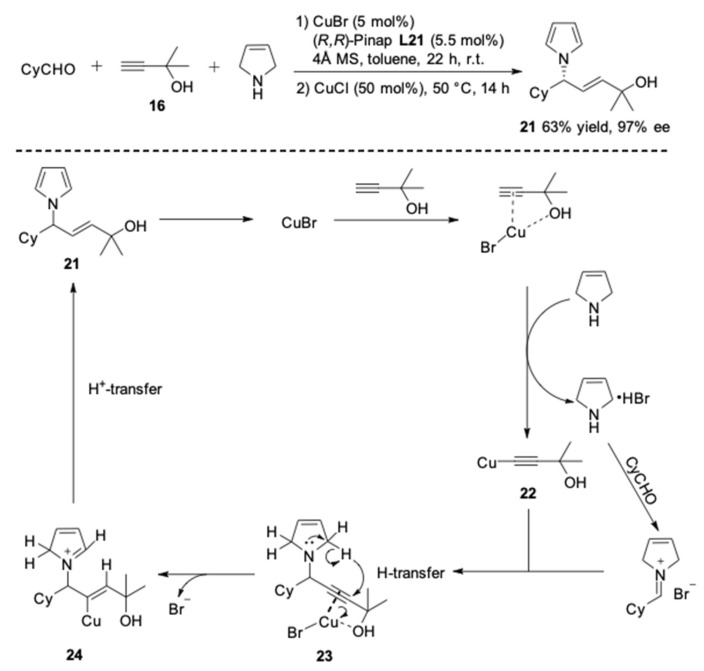
The synthesis of *N*-allyl amines via A^3^-couplings.

Next the amine component was expanded to pyrroline. In this reaction, instead of the normal propargylic amines, optically active *N*-allyl pyrrole **21** was unexpectedly obtained in 97% ee with a complete *E*-stereoselectivity (Figure 21) [64]. Based on the results of deuterium-labeling experiments, a possible mechanism of the coupling-semireduction reaction was proposed in Figure 21. It suggested that the alkyne **16** was coordinated with the copper(I) complex through the hydroxyl oxygen. Then the copper alkynylide specie **22** reacted with the iminium ion, yielding the corresponding propargylic amine-CuBr complex **23**. This compound would afford the iminium intermediate **24** via an anti-1,5-hydride transfer, in which the unsaturated cyclic dialkylamine acted as a hydrogen donor. Finally, the protodemetalation of **24** resulted in the *N*-allyl pyrrole **21** and regenerated the catalyst.

**Figure 22:**
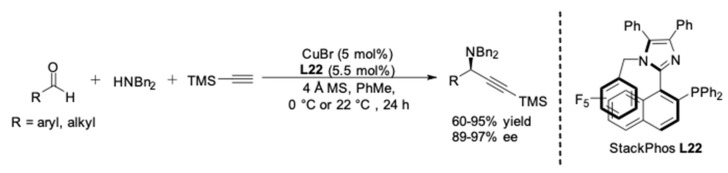
Enantioselective A^3^-coupling employing **L22**.

In 2013, an atropisomeric P,N-ligand **L22**, which they named StackPhos, was designed and synthesized by Aponick and co-workers. Instead of the fused 6-membered aromatics, an imidazole ring was incorporated into the biaryl system (Figure 22) [65]. The N-containing heterocycle not only contains a coordinating atom, but also provides the possibility to stabilize the chiral conformation through π-stacking. Then the performance of **L22** was tested in the AA^3^-coupling reaction of silylacetylene. Delightfully, the reactivity was enhanced compared to Quinap. The corresponding chiral propargylamines were isolated in high yields (60–95%), the reaction time was drastically reduced from 4 days to 24 h with the StackPhos ligand compared with other axially chiral ligands such as Quinap and Pinap. It is noteworthy that the catalyst system enabled the use of both aliphatic and aromatic aldehydes with excellent enantioselectivity (89–97% ee).

Following the same strategy, new P,N-ligands **L23** and **L24** (StackPhim) were prepared by the same group in 2017 [66]. The two diastereomeric phosphines could be readily separated by simple column chromatography without a resolution process. Then the ligands were evaluated in CuBr-catalyzed AA^3^-coupling with butynyl diol ***rac*-25**. As shown in Table 4, the desired propargyl amine **26** was obtained in high yields after 4 h. Moreover, the subsequent Au-catalyzed cyclization of the alkyne provide an efficient access to chiral 2-aminoalkyl furan **27** [67]. The (*S,R,R*)-StackPhim **L23** exhibited superiority in stereocontrol toward this reaction compared with the previous reported (*S*)-StackPhos **L22** (Table 4, entry 2 vs. 1). Notably, the stereoselectivity was further improved to 94% ee with the diastereomeric ligand **L24** (Table 4, entry 3). Once again, the substrate scope of the aldehyde had little influence on both the yields and the stereoselectivities.

**Figure 23:**
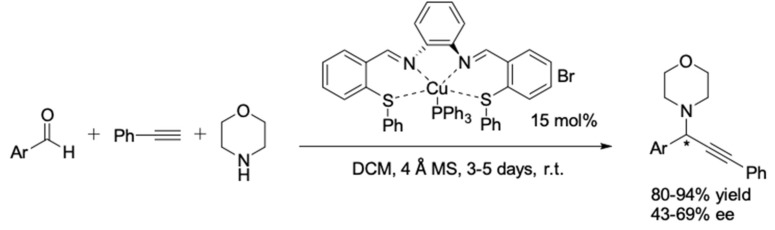
AA^3^-coupling reactions based on Cu(I)/Schiff base complex.

In 2014, Naeimi et al. reported the use of Schiff base as a chiral tetradentate ligand in the AA^3^-coupling reaction of morpholine [68]. The preparation of the catalyst was very simple using readily available chemicals. Nevertheless, it is regrettable that the Cu(I) complex of thiosalen could only deliver the corresponding propargylamines in moderate to good enantiomeric excesses (43–69% ee) (Figure 23).

**Figure 24:**
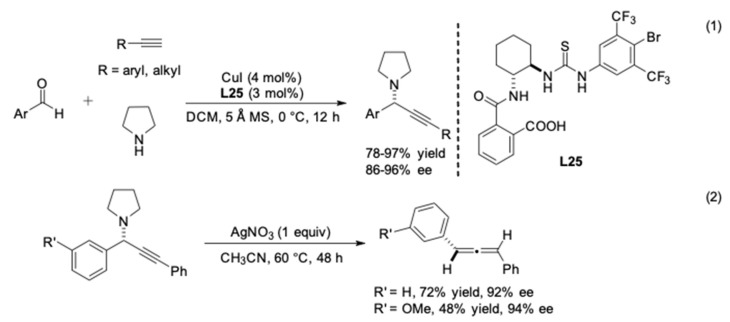
Enantioselective three-component reactions with thiourea and CuI.

In addition to the use of phosphine-based ligands, Seidel reported the application of carboxylic acid-thiourea as a cocatalyst in Cu(I)-catalyzed asymmetric A^3^ reactions with pyrrolidine [69]. A series of urea catalysts were prepared and tested. The authors found that the combination of cocatalyst **L25** and CuI exhibited the best efficiency (Figure 24, Equation (1)). They also attempted to rationalize the roles of the cocatalyst. The data suggested that **L25** acted as a ligand for copper(I), forming a highly reactive copper acetylide complex. However, the role of the carboxylate moiety in the enantiodetermining step remained unclear. In addition, this method was successfully transferred to the synthesis of chiral allenes in the presence of AgNO_3_ (Figure 24**,** Equation (2)) [70].

## 4. Conclusions

In summary, this review has demonstrated the recent advances in asymmetric A^3^-coupling reactions. From the perspective of atom and step economy, the multicomponent character of this process provides a great convenience for the enantioselevtive synthesis of propargylic amines. Although great progress has been made, there are still substantial hurdles to be overcome. For example, the catalytic efficiency needs to be further improved, the substrate scope needs to be expanded, and mechanisms need to be investigated in greater detail. Tandem processes that combine different types of reactions in a single operation are also appealing. It is reasonable to believe that the discovery and development of new chiral ligands will continue to flourish and result in more developments in the years to come.

## Figures and Tables

**Table 1:**
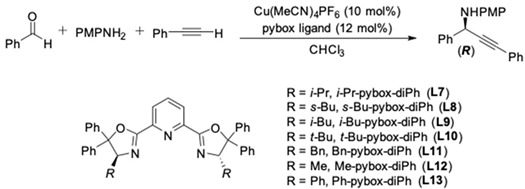
Scope of different pybox on AA^3^-coupling.

Entry	Pybox	Time	Yield ^a^ (%)	ee (%)
1	*i-*Pr-pybox-diPh (**L7**)	16 h	98	90
2	*s-*Bu-pybox-diPh (**L8**)	18 h	97	93
3	*i-*Bu-pybox-diPh (**L9**)	4 days	56	63
4	*t-*Bu-pybox-diPh (**L10**)	22 h	90	68
5	Bn-pybox-diPh (**L11**)	5 days	45	64
6	Me-pybox-diPh (**L12**)	4 days	51	53
7	Ph-pybox-diPh (**L13**)	28 h	96	75(***S***) ^b^

^a^ All the reactions were performed under an argon atmosphere and yields were reported as isolated yield. ^b^ (R,R)–Ligand was used.

**Table 2:**
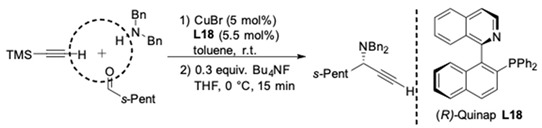
Catalyst recycling on the AA^3^-coupling.

Run	Yield (%) *^a^*	ee (%) *^b^*
1	99	98
2	87	97
3	89	98

*^a^* Isolated yield. *^b^* Enantiomeric excess determined by HPLC using Chiracel OD-H column (*n-*heptane: *i-*PrOH).

**Table 3:**
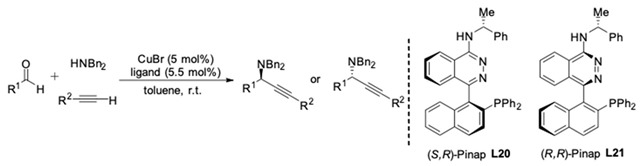
CuBr/Pinap-catalyzed AA^3^-coupling.

R^1^	R^2^	Ligand	Yield [%]	Ee [%]	Quinap [% ee]
*i-*Pr	Me_3_Si	**L20**	84	98 (*R*)	92
		**L21**	82	99 (*S*)	
*i-*Pr	Ph	**L20**	88	90 (*R*)	84
		**L21**	82	95 (*S*)	
*i-*Bu	*i-*Bu	**L20**	74	91 (*R*)	82
		**L21**	72	94 (*S*)	

**Table 4:**
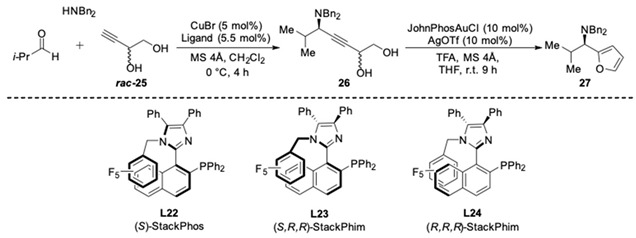
The evaluation of StackPhim.

Entry	Ligand	Product	Yield 26/26′	Yield 27/*ent*-27	ee
1	**L22**	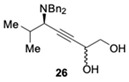	86%	80%	66%
2	**L23**		64%	75%	82%
3	**L24**	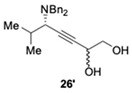	74%	81%	−94%
